# ANG1 treatment reduces muscle pathology and prevents a decline in perfusion in DMD mice

**DOI:** 10.1371/journal.pone.0174315

**Published:** 2017-03-23

**Authors:** Kelly M. Gutpell, Nikola Tasevski, Boaz Wong, William Thomas Hrinivich, Feng Su, Jennifer Hadway, Lise Desjardins, Ting-Yim Lee, Lisa Marie Hoffman

**Affiliations:** 1 Lawson Health Research Institute, London, Ontario, Canada; 2 Department of Anatomy and Cell Biology, University of Western Ontario, London, Ontario, Canada; 3 Department of Medical Biophysics, University of Western Ontario, London, Ontario, Canada; 4 Robarts Research Institute, London, Ontario, Canada; Rutgers University Newark, UNITED STATES

## Abstract

Vascular endothelial growth factor (VEGF) and other pro-angiogenic growth factors have been investigated to enhance muscle tissue perfusion and repair in Duchenne muscular dystrophy (DMD). Current understanding is limited by a lack of functional data following *in vivo* delivery of these growth factors. We previously used dynamic contrast-enhanced computed tomography to monitor disease progression in murine models of DMD, but no study to date has utilized this imaging technique to assess vascular therapy in a preclinical model of DMD. In the current study, we locally delivered VEGF and ANG1 alone or in combination to dystrophic hind limb skeletal muscle. Using functional imaging, we found the combination treatment as well as ANG1 alone prevented decline in muscle perfusion whereas VEGF alone had no effect compared to controls. These findings were validated histologically as demonstrated by increased alpha-smooth muscle actin-positive vessels in muscles that received either VEGF+ANG1 or ANG1 alone compared to the sham group. We further show that ANG1 alone slows progression of fibrosis compared to either sham or VEGF treatment. The findings from this study shed new light on the functional effects of vascular therapy and suggest that ANG1 alone may be a candidate therapy in the treatment of DMD.

## Introduction

Vascular-targeted therapy to treat Duchenne muscular dystrophy (DMD) has been investigated since the early 2000’s [1]. The proposed mechanisms by which angiogenic therapy may alleviate the pathophysiology associated with DMD are numerous. Previous work has provided evidence of compromised vasculature in the disease, including impaired angiogenesis [2] and decreased vascular density in the mdx mouse, the most widely used murine model of DMD [3], as well as in the golden-retriever model of muscular dystrophy [4]. As such, many groups have attempted to increase vascular density in dystrophic muscle by treating it with vascular endothelial growth factor (VEGF), a well-known and potent inducer of angiogenesis [5–7]. Histological markers of endothelial cells, cells that make up the luminal wall of vessels, demonstrate increased vascular density following VEGF treatment. Other studies have also shown that VEGF levels are decreased in some muscle groups in mdx mice as well as in patients [8]. These findings are somewhat inconclusive, though, as others have shown the opposite: that VEGF levels are increased in dystrophic muscle tissue. This discrepancy is likely due to a temporally dependent alteration in expression that differs at various stages of the disease. Interestingly, hypoxia-inducible factor-1 alpha (HIF1-α) is increased in DMD patients [9] and others have shown that increases in HIF1-α in the mdx mouse brain correspond to elevated levels of VEGF [10].

Although promising results have been shown regarding the use of VEGF to alleviate ischemia, there are a number of questions that remain unanswered. First, it has been widely proposed that VEGF, while inducing angiogenesis, creates only immature and “leaky” vessels that do not confer significant functional benefit to the muscle [11]. Although histological analyses have revealed an increase in vascular density following VEGF treatment, whether these newly formed vessels are functional has not been rigorously investigated. Groups have therefore begun to use VEGF in combination with other factors, particularly angiopoietin-1 (ANG1), to induce vascular maturation [12–15].

Binding of ANG1 to the Tie2 receptor on endothelial cells activates the receptor’s kinase activity, producing a cellular response that results in vessel survival and stabilization [16]. Receptor activation increases phosphatidylinositol 3-kinase (PI3K) activity, leading to stimulation of AKT, a cell survival signalling molecule that inhibits transcription factors essential in vascular destabilization [17]. Activation of the PI3K pathway also increases expression of survivin, an inhibitor of apoptosis in endothelial cells [18]. Upon Tie2 activation, vascular-endothelial cadherin increases adhesion between endothelial cells, lending to an increase in overall vessel stability [11,19]. Importantly, ANG1 recruits vascular smooth muscle cells by signalling through endothelial cells [20,21]. This vascular smooth muscle lining ultimately confers functional maturity to newly formed vasculature [22].

Given the role of VEGF and ANG1 in angiogenesis, various groups have attempted to exploit their function as a vascular-targeted approach to treating ischemia in DMD [23]. Indeed, VEGF administration has been shown to increase endogenous repair and enhance the efficacy of transplanted cell populations. Still, questions remain regarding the reality of using these factors to treat DMD. Importantly, very little data exists describing the functional efficacy these factors exert in a longitudinal manner in DMD models. Positron emission tomography demonstrates that blood flow is not affected by VEGF treatment alone in the rat skeletal muscle, but is significantly increased when VEGF is combined with ANG1 [24]. Thus, the objective of the present study is to non-invasively assess the effect of VEGF treatment alone or in combination with ANG1 in dystrophic murine hind limb skeletal muscle. Our group has previously reported the use of dynamic contrast-enhanced computed tomography (DCE-CT) to monitor disease progression in murine models of DMD [25], but no study to date has attempted to assess therapeutic intervention in preclinical studies using this imaging modality. Further, we utilize the mdx/utrn+/- mouse, a model that lacks dystrophin and is heterozygous for utrophin, a dystrophin analogue. Previous studies have suggested the heterozygous mouse as a superior model for DMD research since this mouse develops fibrosis to a greater extent compared to the dystrophin-null mdx mouse [26]. Using the mdx/utrn+/- mouse, which is more prone to fibrosis, is of particular importance given recent findings showing a potential role of VEGF in exacerbating disease severity in other fibrotic diseases such as scleroderma and idiopathic pulmonary fibrosis [27,28]. Additionally, long-term overexpression of VEGF promotes fibrosis in skeletal muscle in the ischemic hind limb rabbit model [29]. Therefore, we sought to determine whether ANG1 alone might be sufficient to slow both the decline in muscle perfusion and progression of fibrosis in the mdx/utrn+/- mouse.

## Materials and methods

### Animal care and ethics statement

Experiments were performed at Lawson Health Research Institute at St. Joseph’s Health Care (SJHC) in London, Ontario. Heterozygous mdx/utrn+/- mice, originally generated by Dr.’s Mark Grady and Josh Sanes (Washington University, St. Louis), were generously provided to us by Dr. Robert Grange (Virginia Polytechnic and State University) and maintained in the Animal Care Facility at SJHC [30]. Colonies were maintained under controlled conditions (19–23°C, 12 hour light/dark cycles) and allowed water and food *ad libitum*. Nine to ten week-old mice were used in this study. All procedures involving animal experiments were carried out in strict accordance with the Canadian Council on Animal Care (CCAC) and were approved by the Animal Use Subcommittee at Western University.

### Genotyping

Genomic DNA from tail snips or ear notch tissue was used for genotyping. Briefly, ear notch tissue was lysed in a proteinase K solution at 50°C overnight. DNA was diluted appropriately and polymerase chain reaction was used to amplify the utrophin gene using Platinum Taq polymerase. Presence of the utrophin gene was detected using the following set of primers (Sigma): 5’-TGCAGTGTCTCCAATAAGGTATGAAC-3’, 5’-TGCCAAGTTCTAATTCCATCAGAAGCTG -3’ (forward primers) and 5’-CTGAGTCAAACAGCTTGGAAGCCTCC-3’ (reverse primer).

### ELISAs

To determine endogenous levels of VEGF and ANG1, we used a Quantikine Mouse VEGF kit (R&D Systems) and a Mouse ANG1 ELISA kit (Lifespan Biosciences). 10 week-old mdx/utrn+/- mice (n = 6) and C57Bl10 (n = 6) were euthanized. Dissected tissue was placed in ice cold PBS, homogenized and stored overnight at -20°C to ensure complete lysis of homogenates. Samples were then centrifuged at 5000xg for 5 minutes and only the supernatant assayed. Total protein was quantified using the bicinchoninic acid Assay (Pierce) prior to ELISA assay. All samples were run in duplicate and absorbance was measured at 450nm.

### Growth factor delivery

Affi-Gel Blue Beads (BioRad) were air-dried in a cell culture hood under sterile conditions overnight. The next day, beads were re-suspended in 10ul of: sterile phosphate-buffered saline (PBS), 1 ug recombinant mouse VEGF, 5 μg recombinant human ANG1, or a combination of VEGF and ANG1. Beads were incubated in the growth factors overnight. The next day, beads were centrifuged for 5 mins at 12,000rpm, the supernatant was removed and the beads were re-suspended in 10μl sterile PBS. Hind limb hair was gently plucked and the exposed skin was wiped with isopropyl alcohol to ensure sterility. The beads were implanted intramuscularly into the posterior compartment of the hind limb (lateral head of gastrocnemius muscle) as follows: mice in the “sham” group received a sham injection (PBS-soaked beads) in both hind limbs, “VEGF” mice received sham injection in the right hind limb and VEGF-coated beads in the left, “VEGF+ANG1” group received VEGF-coated beads in the right hind limb and VEGF+ANG1-coated beads in the left, and “ANG1” mice received sham injection in the right hind limb and ANG1 in the left hind limb. Hind limb hair was gently plucked and the exposed skin was wiped with isopropyl alcohol to ensure sterility. Injections took place before the anatomical axial CT scan while the mouse was anesthetised.

### Dynamic contrast-enhanced computed tomography

Mice were scanned at baseline and 16 days post-injection (time point based on pilot studies previously conducted in our lab). During each imaging session, anaesthesia was induced with 3% isofluorane and maintained with a 1.5% oxygen-balanced isofluorane mixture, delivered at a constant rate of 1L/min. DCE-CT protocol was adapted from a previous study [25]. Briefly, following an anatomical axial scan, each mouse received 200 μL of Conray 43 contrast agent (diluted 1:2 with saline) at an injection rate of 2.0 ml/min with an infusion pump (New Era Pump Systems Inc) via tail vein catheter. CT Perfusion software (GE Healthcare) was used to quantify blood flow (BF) and blood volume (BV) based on functional maps from the acquired series of CT images. Perfusion data acquired over the course of the study was normalized with respect to baseline values for each mouse to minimize biological variability between mice, thus allowing us to assess longitudinal changes between groups. Regions of interest were drawn around the whole cross-sectional slice of the hind limb, excluding the tibia and fibula, and three slices covering the leg were included in each calculation.

### Tissue preparation

At the end of the imaging study, mice were sacrificed via gas euthanasia followed by cervical dislocation. Gastrocnemius (GM) muscles were dissected, immediately fixed in formalin, and embedded in paraffin. Extreme care was taken to ensure muscles were embedded in the same orientation across each muscle group. Tissue blocks were sectioned at 5um thickness and dried at 37°C overnight. To achieve representative sections from the whole muscle tissue, serial sections were taken every 30 slices. Tissue sections were then deparaffinised and rehydrated in a series of xylene and ethanol washes to prepare them for subsequent Masson’s trichrome staining for collagen content (performed at the Pathology Department at University Hospital, London, ON).

### Immunohistochemistry

Tissue sections were processed for immunocytochemistry by deparaffinising and rehydrating sections followed by heat-mediated antigen retrieval in a citrate buffer for 20 minutes. Slides were then cooled slowly to room temperature and incubated in blocking buffer (1% BSA, 10% goat serum in PBS) for one hour. Sections were incubated with anti-α-SMA (Abcam, 1:200) or anti-CD31 (Abcam, 1:50) primary antibodies at 4°C overnight. Following thorough washing with PBS, Alexafluor IgG (Life Technologies, 1:1000) secondary antibodies were used to visualize the primary antibodies and immersion in 0.1% Sudan Black B was performed to quench autofluorescence. Finally, ProLong Gold anti-fade with DAPI (Life Technologies) was added to visualize the nuclei and to mount the coverslips onto glass slides. Fluorescent images were acquired on a Nikon Eclipse microscope.

### Microscopy and image analysis

For Masson’s trichrome sections, colour histological images were acquired on a Zeiss Axioscope microscope using Northern Eclipse Imaging software. Non-overlapping fields of view of the entire tissue were taken for each section. Collagen content was assessed across the entire tissue slice and automatically quantified using an in-house colour thresholding algorithm written in MATLAB 2015b (Mathworks, Natick, MA, USA) designed to separate red and blue image components as previously described [31]. The percentage of each slide area positive for collagen presence was calculated, and automatic thresholds were manually verified with labeled colour overlays on the original histology images to ensure that collagen presence was accurately identified.

For αSMA and CD31 sections, grey scale fluorescence images were acquired on a Nikon Eclipse Microscope using NIS Elements Microscope Image Softare. Non-overlapping fields of view of the entire tissue were taken for each section. A semi-automatic grey scale thresholding algorithm was implemented in MATLAB 2015a to quantify the area of each slide positive for αSMA or CD31 while mitigating variations due to image exposure and auto-fluorescence; referred to as “background” intensity variations. The three major steps of the algorithm are 1) background intensity estimation, 2) stain identification, and 3) stain area calculation. These steps are briefly described as follows. 1) The intensity gradient magnitude of each slide was calculated, and contiguous regions with a gradient magnitude less than a constant threshold were assigned as background. This background threshold was empirically selected as 40 intensity units, or roughly 8% of the maximum signal intensity gradient observed across slides. 2) All closed non-background regions with maximum signal intensity greater than a stain threshold were assigned positive for stain presence. The stain threshold was manually selected as either 4× or 5× times the mean background signal intensity of each slide. 3) Within each closed non-background region considered positive for stain presence, the final stained area was determined by applying a threshold of 25% of the maximum intensity in that region. The percentage of total area positive for stain was calculated for each slide. Again, automatic thresholds were manually verified using labelled colour overlays on the original images, and any features incorrectly identified as positive for stain were manually edited.

### Statistical analysis

All analyses were performed using Graphpad Prism software. Data results are expressed as mean ± standard deviation (SD). Comparison between groups was performed using a Wilcoxon test or two-tailed t-test, as appropriate. When more than two groups were present, a one-way analysis of variance (ANOVA) was performed adjusting for multiple comparisons as appropriate. P value of less than 0.05 was considered significant. Replicate numbers are indicated in the figure legends.

## Results

### Endogenous levels of both VEGF and ANG1 are significantly reduced in severely fibrotic diaphragm tissue, but not weakly affected Gastrocnemius (GM) tissue of the mdx/utrn+/- mouse

**Fig 1:**
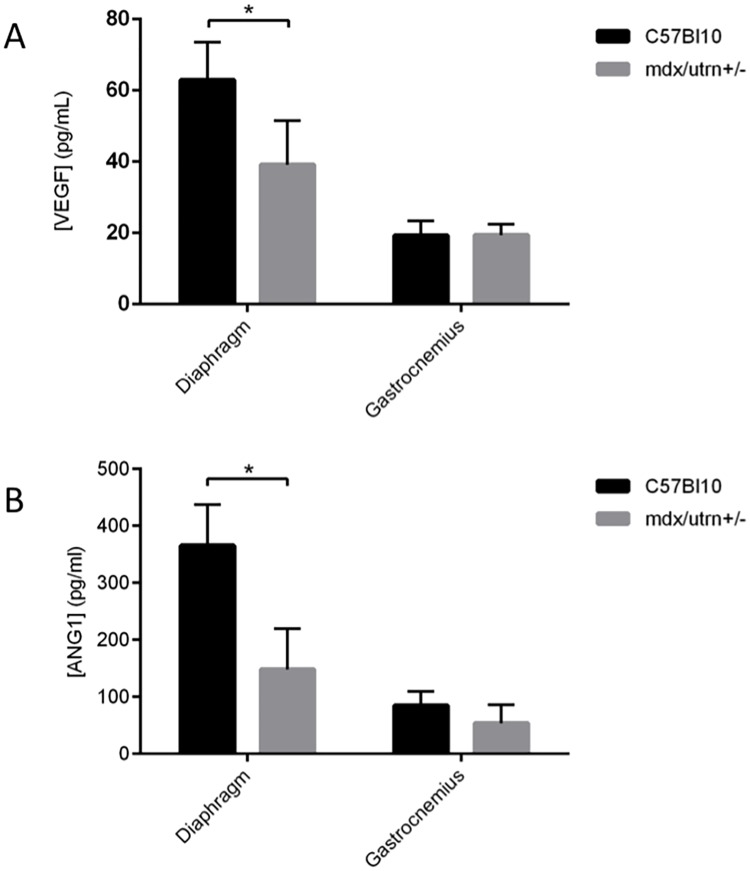
VEGF and ANG1 are decreased in dystrophic diaphragm and gastrocnemius murine muscles. ELISA analysis of VEGF and ANG1 in 9 to 10 week-old mdx/utrn+/- diaphragm and GM muscles compared to healthy wild type controls. A. VEGF was lower in mdx/utrn+/- diaphragm muscles compared to healthy wild-type controls. VEGF expression was not significantly different between dystrophic and healthy GM muscles. B. ANG1 was lower in mdx/utrn+/- diaphragm muscles compared to healthy wild-type controls. ANG1 expression was not significantly different between dystrophic and healthy GM muscles. n = 6 per group, *P < 0.05, by Student’s t-test. Error bars represent SD.

Endogenous expression of VEGF and ANG1 was measured in the diaphragm and GM muscles at 9–10 weeks of age (Fig 1). The level of VEGF in GM muscle of dystrophic mice (19.6pg/ml) was similar to that measured in wild-type mice (19.3pg/ml, p = 0.93). However, compared to healthy diaphragm muscle (63.0pg/ml), the concentration of VEGF in dystrophic diaphragm muscle (39.2pg/ml) was significantly reduced (p = 0.0049). A similar trend was observed for the endogenous concentration of ANG1 in healthy versus dystrophic muscles. Specifically, ANG1 expression in healthy and dystrophic GM muscles was 85.3 and 54.1 pg/ml, respectively, but these values were not determined to be significantly different from one another (p = 0.09). In contrast, a marked reduction of ANG1 expression was measured in the diaphragm of dystrophic mice relative to wild-type controls (148.3 and 365.6 pg/ml, respectively; p<0.0001)

### Effect of angiogenic growth factors on perfusion

**Fig 2:**
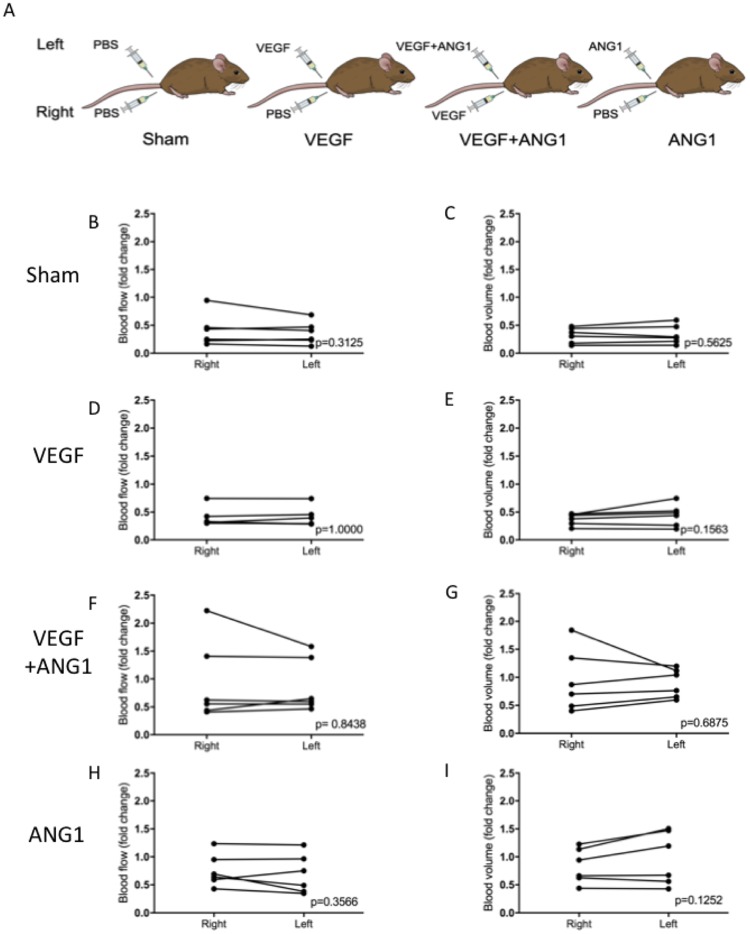
Perfusion measured at endpoint is not significantly different between hind limbs, regardless of treatment. (A) Schematic representation of treatment groups. “Sham” group mice received sham injections in both hind limbs. “VEGF” mice received sham injection in the right hind-limb, VEGF in the contralateral limb. “VEGF+ANG1” mice received VEGF in the right hind-limb and VEGF+ANG1 in the contralateral limb. “ANG1” mice received ANG1 in the left hind limb and a sham injection in the contralateral limb. (B) Blood flow (left) and blood volume (right) did not differ between hind limbs, allowing for perfusion measurements to be assessed based on the averaged BF and BV following treatment. n = 6, P < 0.05, by Wilcoxan signed-rank test.

**Fig 3:**
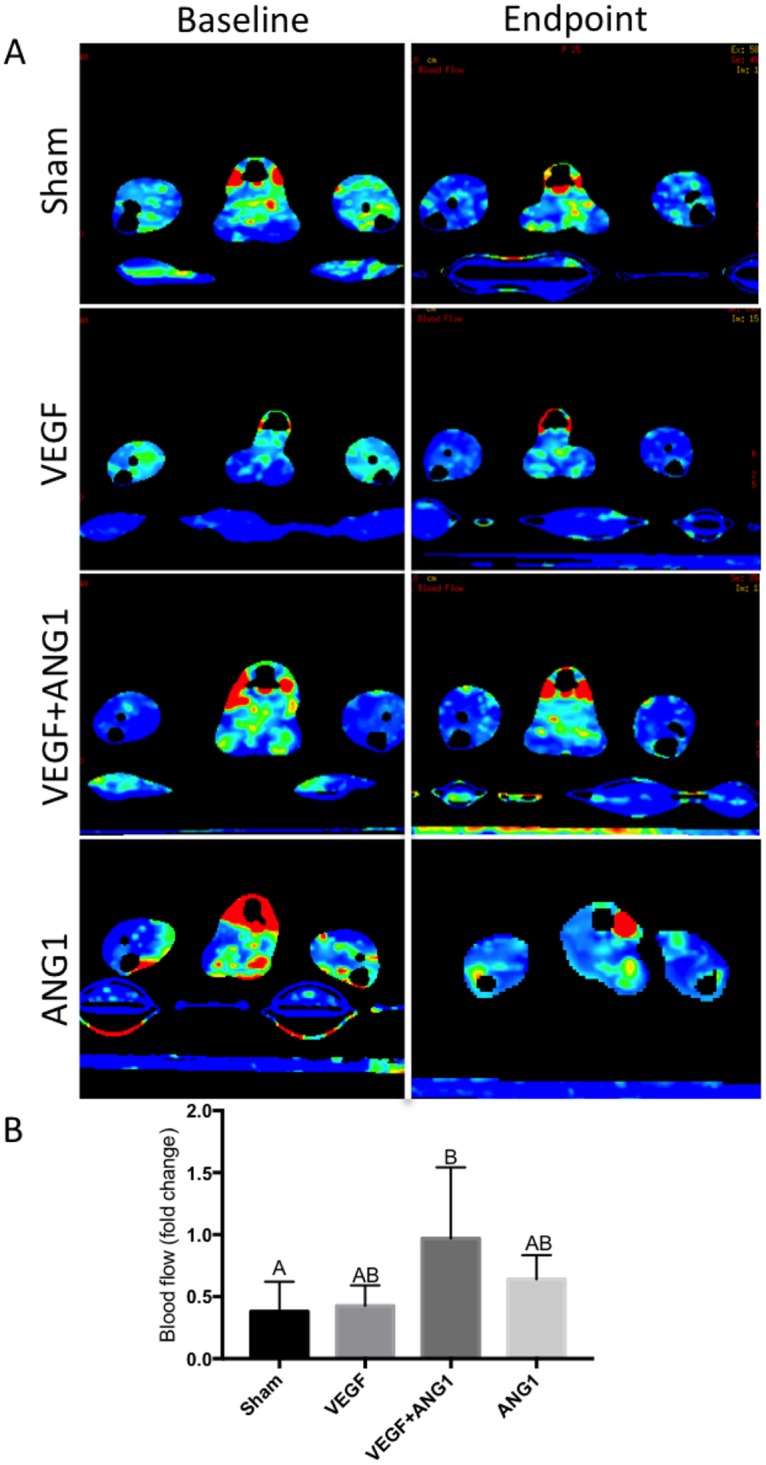
VEGF+ANG1 slows decline in hind limb muscle perfusion blood flow 16 days post-treatment in mdx/utrn+/- mice. A. Representative DCE-CT blood flow maps of sham-injected, VEGF-, VEGF+ANG1-, and ANG1-treated hind limbs. B. Blood flow decreased in all but two mice over the course of the study. n = 6, P < 0.05, by one-way ANOVA. Error bars represent SD. Means with different letters are significantly different.

**Fig 4:**
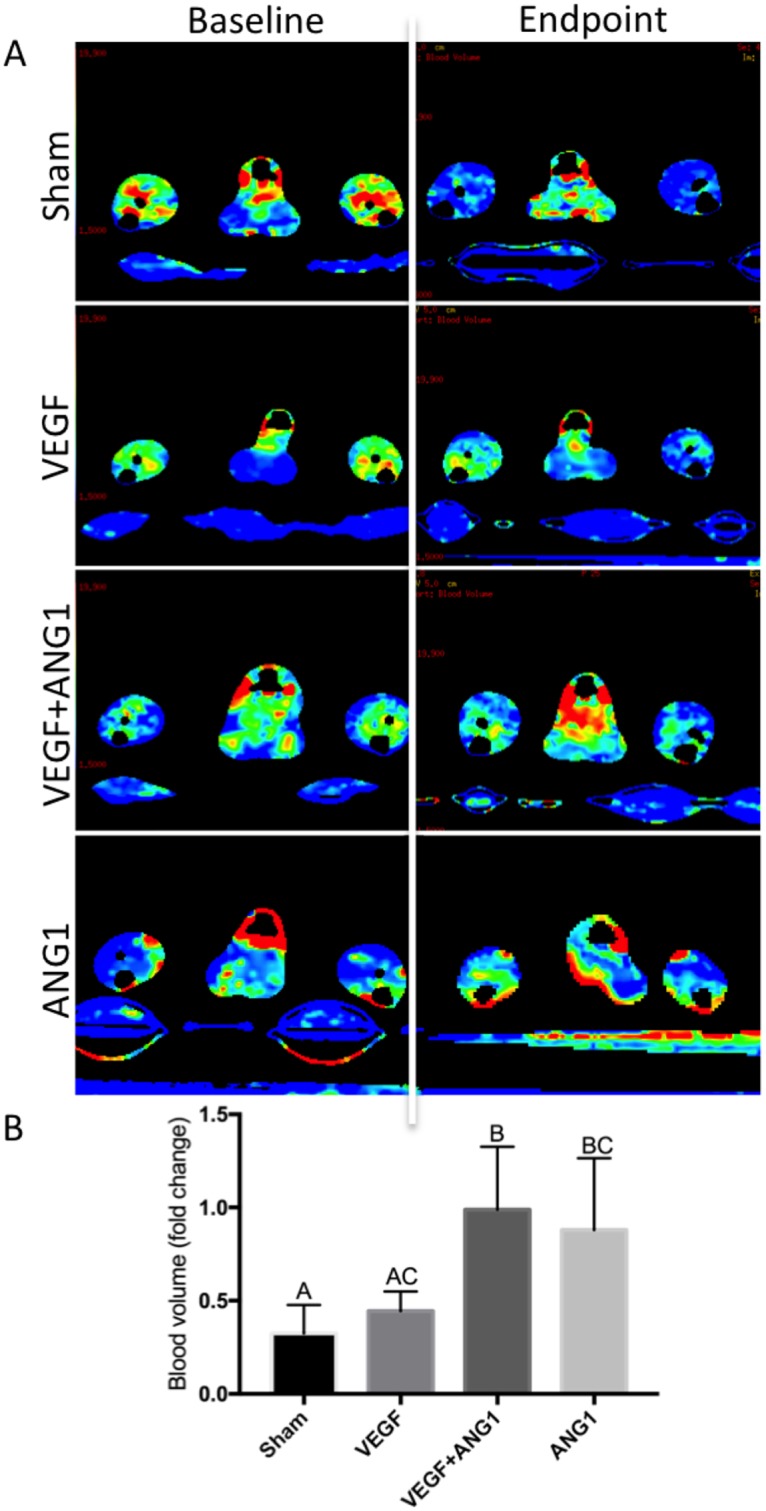
Both VEGF+ANG1 and ANG1 treatment prevent decline in blood volume 16 days post-treatment in mdx/utrn+/- hind limb skeletal muscle. A. Representative DCE-CT blood volume maps of sham-injected, VEGF-, VEGF+ANG1-, and ANG1-treated hind limbs. B. Blood volume was not significantly different in sham-injected and VEGF-treated hind limbs. VEGF+ANG1 treatment resulted in significantly higher fold-change in blood volume compared to both VEGF and sham group. n = 6, P < 0.05, by one-way ANOVA. Error bars represent SD. Means with different letters are significantly different.

Blood flow (BF) and blood volume (BV) were assessed as parameters of perfusion in this study. There was no significant difference measured between the two hind limbs, regardless of treatment, indicating systemic delivery of the growth factors through intramuscular injection. BF and BV are therefore presented as fold change averages between the two limbs after 16 days of growth factor treatment (Fig 2). Absolute BF and BV values were compared between treatment groups. Neither parameters were significantly different between treatment groups for BF (p = 0.0715) or BV (0.0819, Table 1). An overall decrease in BF was observed in mice of all treatment groups over the course of the study (Fig 3). There was no significant difference in BF fold change between mice in the sham-injected and VEGF-treated groups (p = 0.9951). Additionally, ANG1 did not result in any differences compared to the sham (p = 0.5481) and VEGF group (p = 0.6911). BF fold change was significantly higher in the VEGF+ANG1-treated mice compared to the sham group (p = 0.0306). Overall, the effect of growth factor treatment was more marked in BV changes. Changes in BV did not differ between the sham group and the VEGF-treatment group (p = 0.8727). Both the combination VEGF+ANG1 treatment as well as the ANG1 treatment alone resulted in significantly higher BV fold change compared to the sham group (p = 0.0022 and p = 0.0107, respectively). Additionally, VEGF+ANG1 treatment led to higher BV fold change than VEGF alone (p = 0.0121, Fig 4).

**Table 1:** Mean absolute values (mean ± SD) of Blood Flow (BF) and Blood Volume (BV) for each experimental group at baseline. P-values to the right of each mean column indicate that no significant difference existed between treatment groups at baseline. n = 6, P < 0.05, by one-way ANOVA.

Treatment	Absolute BF ± SD	BF p-value	Absolute BV ± SD	BV p-value
**Sham**	58.33 ± 23.17	0.0715	6.26 ± 1.63	0.0819
**VEGF**	51.60 ± 8.25		4.67 ± 2.22	
**VEGF+ANG1**	37.46 ± 9.48		3.59 ± 1.49	
**ANG1**	40.68 ± 7.44		5.58 ± 1.00	

### ANG1 treatment induces vessel maturation in mdx/utrn+/- hind limb muscle

**Fig 5:**
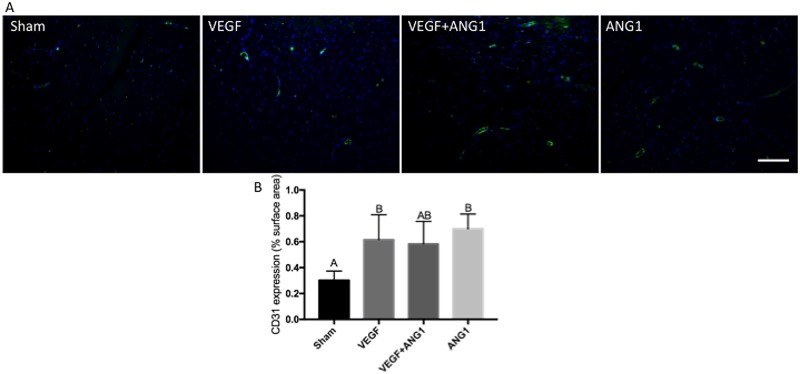
VEGF and ANG1 increase vascular density following localized delivery in the gastrocnemius muscle of mdx/utrn+/- mice. A. Representative images of CD31 expression (green) in gastrocnemius muscles of mdx/utrn+/- following 16 days of angiogenic growth factor treatment. DAPI was used as a counterstain (blue). Scale bar = 100μm. B. Quantification of CD31 expression in the four treatment groups. n = 4, p<0.05 by one-way ANOVA. Error bars represent ± SD and means with different letters are significantly different.

**Fig 6:**
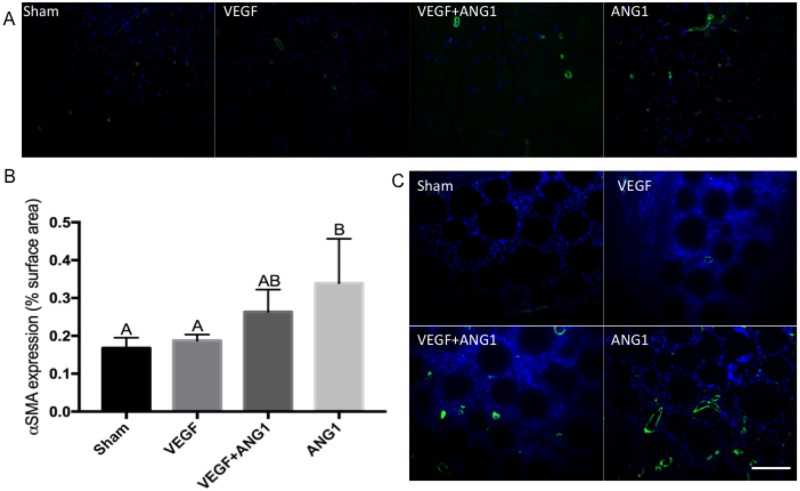
ANG1 treatment increases vessel maturation following treatment in mdx/utrn+/- GM muscle. A. Representative immunofluorescence images of αSMA expression (green) in sham-injected, VEGF- and VEGF+ANG1-treated GM muscles. B. Immunohistochemical analysis of αSMA-positive vessels, represented as percent image area. n = 4 for all groups, by one-way ANOVA. Error bars represent SD and means with different letters are significantly different. C. Growth factor-coated beads are visible at the injection site. αSMA-positive vessels are detected at the injection site of VEGF+ANG1- and ANG1-treated hind limbs, and absent at the injection site of sham-injected and VEGF-treated hind limbs. DAPI was used as a counterstain. Scale bar = 100 μm.

Expression of CD31 was used to assess vascular density in hind limb muscle following treatment (Fig 5). Both VEGF- and ANG1-treated hind limbs had significantly greater CD31 expression compared to sham-injected controls (p = 0.0452 and p = 0.0109). The difference in CD31 expression between the sham group and VEGF+ANG1group was not significant (p = 0.0780). Since CD31 marks only endothelial cells, including those in immature, leaky vasculature, we further employed IHC analysis to measure alpha-smooth muscle actin (αSMA) expression in mature vessels (Fig 6). No significant differences were measured between sham-injected and VEGF-treated mice (p = 0.9765) or between sham and VEGF+ANG1 (p = 0.2400). Mice treated with ANG1 alone, however, showed significantly greater αSMA-positive vessels in hind limb muscles compared to either the sham (p = 0.0170) or VEGF groups (p = 0.0345). Many newly formed αSMA-positive vessels were detected at the injection site in both VEGF+ANG1 and ANG1-treated hind limbs. Very few mature vessels were observed at the injection site of VEGF-treated hind limbs.

### ANG1 decreases collagen deposition in mdx/utrn+/- hind limb muscle

**Fig 7:**
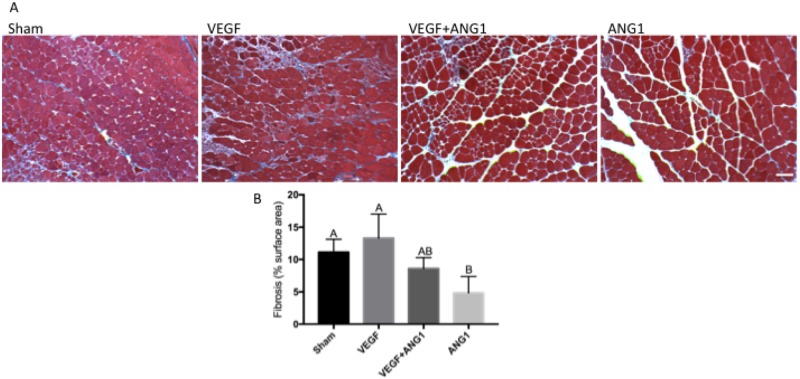
ANG1 treatment decreases collagen deposition following treatment in mdx/utrn+/- GM muscle. A. Representative Masson’s trichrome-stained tissue sections of sham-injected, VEGF-, VEGF+ANG1, and ANG1-treated GM muscles. Collagen deposition appears blue. Scale bar = 100 μm B. Quantification of collagen deposition represented as percent image area. n = 4 for all groups P < 0.05 by one-way ANOVA. Error bars represent SD and means with different letters are significantly different. Scale bar = 100 μm.

To assess the effect of growth factor treatment on muscle fibrosis, we measured collagen deposition using Masson’s trichrome stain (Fig 7). Neither the VEGF treatment alone nor the combination treatment of VEGF+ANG1 affected fibrosis in hind limb muscles when compared to the sham control (p = 0.6369 and 0.5368, respectively). In contrast, ANG1 treatment resulted in less collagen deposition compared to either the sham-injected controls (p = 0.0226) or the VEGF alone group (p = 0.0028).

## Discussion

Vascular endothelial growth factor (VEGF) and angiopoietin-1 (ANG1) are increasingly being considered for their potential role in slowing disease progression in patients with Duchenne muscular dystrophy (DMD) [32]. While prior studies suggest a potential role for these factors in enhancing endogenous repair and cell therapy, studies remain to directly investigate the effects of either growth factor on functional perfusion in a non-invasive manner. Therefore, in the present study, we utilized the mdx/utrn+/- mouse, a murine DMD model more prone to fibrosis than the commonly used mdx mouse, to measure functional perfusion via dynamic contrast-enhanced computed tomography (DCE-CT). The short-term effect of VEGF, ANG1, or a combination of the two was assessed following a low dose, localized delivery for 16 days. Given the high degree of variability between animals with respect to baseline perfusion parameters, the ability to monitor vascular-targeted therapy over time in the same animal is particularly valuable. DCE-CT is a safe and effective means to monitor both disease progression and therapeutic efficacy and shows promise for scaling preclinical studies directly to patients [33,34]. Importantly, we provide the first evidence that VEGF alone is not sufficient to affect functional perfusion parameters in the hind limb skeletal muscle at the dose and duration investigated. Interestingly, we report here that ANG1 alone treatment is sufficient to affect functional perfusion, as demonstrated by a maintained blood volume compared to controls. To our knowledge, this is the first report of ANG1 alone having a significant, functional effect on perfusion *in vivo* in a murine model of DMD. While these findings are promising, they are not surprising given its role in promoting vascular maturity and satellite cell self-renewal [35]. As well, research in other vascular-related diseases such as cardiac ischemia and sepsis has uncovered the deleterious effect that low circulating levels of ANG1 may play in these states [36,37]. In human microvascular endothelial cells (HMVECs), serum from sepsis patients induced intercellular gap formation, and this effect was reversed by supplementation with ANG1 [38].

The findings reported here also highlight the importance of investigating perfusion parameters other than blood flow alone, since the physiological variability of this measurement can overshadow key observations. Blood flow is subject to a number of environmental cues that vastly change flow measurements, such as temperature, fasting, exercise, and stress. While blood volume may also be affected by these factors, it is a more stable function that has the ability to represent changes in the intravascular compartment, or the space that can be perfused during the flow of blood.

The findings from this study highlight the importance of considering the stage of disease progression in assessing vascular therapy. During the first weeks of life, murine models of DMD display classic signs of rapid degeneration and regeneration, accompanied by a robust inflammatory response. This phase is accompanied by a transient increase in perfusion as assessed by DCE-CT [25]. By nine-ten weeks of age, the disease evolves to a more degenerative state and fibrosis begins to predominate, coinciding with a progressive decrease in perfusion. Having critically identified a window of opportunity to intervene with therapeutics, the present study aimed to assess the ability of a combination of VEGF and ANG1 to slow decline in muscle tissue perfusion as well as fibrosis in DMD mice. Our data on endogenous expression of the two growth factors further points to differences in the vasculature at different phases of disease progression. Although we did not measure differences in either growth factor in the hind limb (gastrocnemius) muscle, there was a significant reduction in both VEGF and ANG1 in the diaphragm. Since the diaphragm develops overt fibrosis and muscle degeneration much earlier than hind limb muscles, these differences may point to possible differences in expression of VEGF and ANG1 in the hind limb at later time points. Overall, having well-defined margins between the different disease states could reveal valuable information regarding the effects of VEGF and ANG1 on perfusion, vascular permeability, and fibrosis.

Prior studies have suggested that VEGF treatment decreases fibrosis [7,39], whereas we report no change in collagen deposition following VEGF treatment and in fact report higher levels of fibrosis in this group when compared to the ANG1-treated group. This finding is in line with our previous work showing that VEGF induces stress fiber formation in fibroblasts derived from the GM and diaphragm muscles of mdx/utrn+/- mice. Importantly, studies in other fibrotic diseases including idiopathic pulmonary fibrosis and scleroderma have shown that VEGF exacerbates disease pathology [27,28]. Still, previous work in the DMD field has pointed to an anti-fibrotic role of VEGF. A few variables could account for this discrepancy. The use of the mdx/utrn+/- mouse rather than the mdx mouse, which our group has validated as a more suitable model due to its increased development of fibrosis, may be more responsive to VEGF than its mdx counterpart. This hypothesis speaks to the seed and soil theory whereby the mdx/utrn+/- tissue may be “primed” to respond to VEGF and develop fibrosis, relative to the mdx mouse. Another important consideration that may account for the differences between our findings and those of others with respect to fibrosis is the disease stage assessed in the current study. A number of studies use either young (5 to 7 week-old) or aged (6 month old) mdx mice [6]. It is therefore possible that VEGF plays a reduced pro-fibrotic role in both the early phase of the disease when acute inflammation predominates and in later phases of the disease when overt fibrosis has occurred in the muscle. Lastly, the dose used in this study is much lower than some doses cited in previous studies and may account for differential effects on collagen deposition following treatment. Regardless, a study in rabbit skeletal muscle has also indicated that long-term delivery of VEGF increases collagen deposition. Cumulatively, these findings suggest that the effect of VEGF on fibrosis may significantly impede its use as a therapeutic factor in DMD.

There is a critical need to detect disease changes such as decline in muscle perfusion in early stages so that therapies can be developed before damage, ie., fibrosis, is extensive and irreversible. Advanced non-invasive imaging technologies have immense potential to achieve this; we have used dynamic contrast-enhanced computed tomography (DCE-CT) and positron emission tomography (PET) to identify transient “spikes” in perfusion and ^18^F-fluorodeoxyglucose (^18^F-FDG) uptake, respectively, in the hind limb muscles of preclinical mouse models of DMD [25]. Intensity of these spikes correlates with disease severity, degree of inflammation, and development of muscle fibrosis. Importantly, these studies identified a window of opportunity to intervene with therapeutics aimed at slowing/attenuating the disease process, as demonstrated in the present study. Further use of these technologies to delineate how vascular therapies augment either endogenous muscle repair or cell replacement therapy represent an innovative and critical approach to the treatment of muscle degenerative disorders.

Future directions will aim to develop methods to better control the delivery of angiogenic factors. Although it is well accepted that cell-based delivery systems effectively deliver high payloads, there remains concerns regarding their potential pro-tumorigenic side effects, particularly with regards to VEGF administration [40]. Additionally, although we focused on the hind limb muscles in the current study, vascular therapy will need to be effective in other muscles involved in disease progression, particularly the diaphragm and cardiac muscles. Indeed, fibrosis and degeneration in these tissues account for a majority of fatalities in DMD, and therefore any promising treatment will need to affect these muscles as well. Prior studies investigating the therapeutic efficacy of ANG1 have focused on its effects as a combination treatment with VEGF. The present study suggests that ANG1 alone may improve blood volume, enhance blood vessel maturation, and decrease fibrosis. Overall, these findings support further investigation for the use of ANG1 in the long-term management of DMD.
